# Asia‐Pacific consensus recommendations on the management of generalized pustular psoriasis

**DOI:** 10.1111/1346-8138.17471

**Published:** 2024-10-10

**Authors:** Siew Eng Choon, Peter Anthony Foley, Pravit Asawanonda, Hideki Fujita, Seong‐Jin Jo, Yu‐ling Shi, Colin Theng, Azura Mohd Affandi, Chul Hwan Bang, Maria Lorna Frez, Huang Yu Huei, Doanh Le Huu, Tae‐Gyun Kim, Akimichi Morita, Hazel H. Oon, Pablo Fernández‐Peñas, Natta Rajatanavin, Suganthy Robinson, Latha Selvarajah, Tsen‐Fang Tsai

**Affiliations:** ^1^ Clinical School Johor Bahru, Jeffrey Cheah School of Medicine & Health Sciences Monash University Johor Bahru Malaysia; ^2^ The University of Melbourne, St Vincent's Hospital Melbourne Fitrozy Victoria Australia; ^3^ Skin Health Institute Carlton Victoria Australia; ^4^ Division of Dermatology Chulalongkorn University Bangkok Thailand; ^5^ Division of Cutaneous Science, Department of Dermatology Nihon University School of Medicine Tokyo Japan; ^6^ Department of Dermatology Seoul National University College of Medicine Seoul Republic of Korea; ^7^ Department of Dermatology Shanghai Skin Disease Hospital, School of Medicine, Tongji University Shanghai China; ^8^ Institute of Psoriasis, School of Medicine, Tongji University Shanghai China; ^9^ The Skin Specialists & Laser Clinic Singapore Singapore; ^10^ Department of Dermatology Hospital Kuala Lumpur Kuala Lumpur Malaysia; ^11^ Department of Dermatology Seoul St. Mary's Hospital, College of Medicine, the Catholic University of Korea Seoul Republic of Korea; ^12^ Department of Dermatology St Luke's Medical Center Quezon City Philippines; ^13^ Department of Dermatology Chang Gung Memorial Hospital Taipei Taiwan; ^14^ School of Medicine, Chang Gung University Taipei Taiwan; ^15^ National Hospital of Dermatology and Venereology Hanoi Vietnam; ^16^ Department of Dermatology Cutaneous Biology Research Institute, Severance Hospital, Yonsei University College of Medicine Seoul Republic of Korea; ^17^ Department of Geriatric and Environmental Dermatology Nagoya City University Graduate School of Medical Sciences Nagoya 467‐8601 Japan; ^18^ National Skin Centre and Skin Research Institute of Singapore (SRIS) Singapore Singapore; ^19^ Yong Loo Lin School of Medicine, National University of Singapore Singapore Singapore; ^20^ Department of Dermatology Westmead Hospital Westmead New South Wales Australia; ^21^ Sydney Medical School, Faculty of Medicine and Health, the University of Sydney Westmead New South Wales Australia; ^22^ Phototherapy Unit, Division of Dermatology, Department of Medicine Ramathibodi Hospital, Mahidol University Bangkok Thailand; ^23^ Department of Dermatology Sultan Ismail Hospital Johor Bahru Malaysia; ^24^ Department of Dermatology National Taiwan University Hospital Taipei Taiwan

**Keywords:** Asia‐Pacific, consensus, Delphi panel, generalized pustular psoriasis, management

## Abstract

Generalized pustular psoriasis (GPP) is a rare, chronic, heterogeneous, and potentially life‐threatening disease characterized by primary, sterile, and macroscopically visible pustules with or without systemic symptoms. There are ethnic differences in the genetic mutations associated with GPP that might affect the clinical manifestations and treatment responses. Currently, there is limited evidence from the patient population in the Asia‐Pacific (APAC) region, resulting in a general paucity of information on the effective management of patients with GPP in this region. This modified Delphi panel study aimed to identify current evidence and gain advanced insights to facilitate the development of a regionally tailored APAC consensus on the management of GPP. A systematic literature review (SLR) was conducted to identify published literature and develop consensus statements on (i) definition and clinical course, (ii) diagnosis of GPP, (iii) treatment outcomes, goals, and monitoring measures, and (iv) optimal management strategies and clinical practices. Statements were rated by a panel of dermatologists in two rounds, with the threshold for consensus at ≥80% agreement. Twenty experts from the APAC region reached consensus on 106 statements that were developed based on the SLR and experts' collective expertise. The experts agreed that GPP is a rare, severe, and potentially life‐threatening condition that is distinct from plaque psoriasis. This consensus emphasized the importance of a tailored treatment strategy taking into account the GPP flare severity and each patient's unique clinical circumstances. The experts reached consensus on the severity classification of GPP flares and recommended first‐line and maintenance treatment options for adult GPP, childhood GPP, and GPP in pregnancy. These consensus outcomes have been synthesized into treatment algorithms to guide dermatologists in the APAC region in their clinical decision‐making processes.

## 1 INTRODUCTION

Generalized pustular psoriasis (GPP) is a rare, chronic, and potentially life‐threatening inflammatory disease characterized by recurrent, sudden flares of widespread painful erythema studded with sterile pustules, often accompanied by systemic inflammation.^1^, ^2^, ^3^, ^4^, ^5^, ^6^, ^7^ Reported prevalence rates vary significantly due to differences in study populations, designs, and settings, and variations in data sources, case definitions, and diagnostic criteria.^8^, ^9^, ^10^, ^11^, ^12^, ^13^, ^14^, ^15^, ^16^, ^17^, ^18^, ^19^, ^20^ Early hospital surveys reported prevalence rates of approximately 2 per million in France^8^ and 7 per million in Japan,^13^ which suggested a potentially higher prevalence in Asia. However, recent studies utilizing electronic databases reveal significant variability in prevalence rates. Current figures include 7–9 per million in Brazil,^10^ 90 in the USA,^14^ 30 in England,^18^ 45 in France,^19^ 15 in Sweden,^12^ 111 in Denmark,^20^ 140 in Germany,^14^ 14 in China,^17^ 20–30 in Japan,^14^ 124 in South Korea,^11^ and 198 in Malaysia (higher in Chinese individuals [271] than in Malay [186] or Indian [179] populations).^9^ These variations highlight the need for caution when comparing prevalence rates across different regions.

Interleukin (IL)‐36 is the key driver of disease pathology. The importance of IL‐36 signaling was highlighted by the identification of *IL36RN* mutations almost simultaneously in nine Tunisian families with familial GPP and in three out of five unrelated patients with sporadic GPP in 2011.^21^, ^22^ Subsequently, *IL36RN* disease alleles were described in various ethnic groups,^23^, ^24^, ^25^, ^26^, ^27^, ^28^, ^29^, ^30^ with the highest prevalence in Taiwanese patients with GPP at 75%.^30^ The most common *IL36RN* variant in Asia is c.115+6T>C, whereas p.Ser113Leu is the most common variant in Europe.^23^ Ethnic and geographical variations, including the prevalence of specific variants, suggest diverse disease patterns globally.

A systematic review of the clinical features and genetic status of 233 patients with GPP in 2015 found that *IL36RN* mutations define a severe GPP phenotype characterized by a clinical triad of (i) early disease onset, (ii) high risk of systemic inflammation, and (iii) low prevalence of plaque psoriasis.^26^ This study also showed that heterozygous mutations confer a substantial increase in disease risk in most ethnic groups.^26^ Multiple subsequent studies confirmed that *IL36RN* mutations are associated with a more severe phenotype characterized by early disease onset,^24^, ^25^, ^29^, ^30^ systemic inflammation,^26^, ^28^ and frequent GPP flares.^24^, ^25^, ^27^ Of note, many of the *IL36RN* mutations are single nucleotide polymorphisms and do not cause functional impairment;^30^, ^31^ as such *IL36RN* variants have been reported in up to 10% of control populations. This, together with disease manifestations in patients with monoallelic variant and variation in disease severity among siblings with identical mutations, suggests that environmental triggers or mutations in additional genes may contribute to the complete manifestation of the disease.^23^, ^27^, ^31^

While the recently published global Delphi consensus on the diagnosis, clinical course, treatment goals, and management of GPP provides a valuable foundation,^5^ regional differences necessitate a specific focus on the Asia‐Pacific (APAC) region. Environmental, genetic, and lifestyle factors contribute to variations in GPP epidemiology and presentation. Additionally, cultural beliefs, patient preferences, ethical considerations, and treatment accessibility vary across regions, impacting treatment decisions. An APAC consensus is crucial for addressing accessibility and affordability, and optimizing therapeutic approaches. The aim of this study was to develop an APAC consensus on the management of GPP by utilizing the modified Delphi method.

## 2 MATERIALS AND METHODS

**FIGURE 1:**
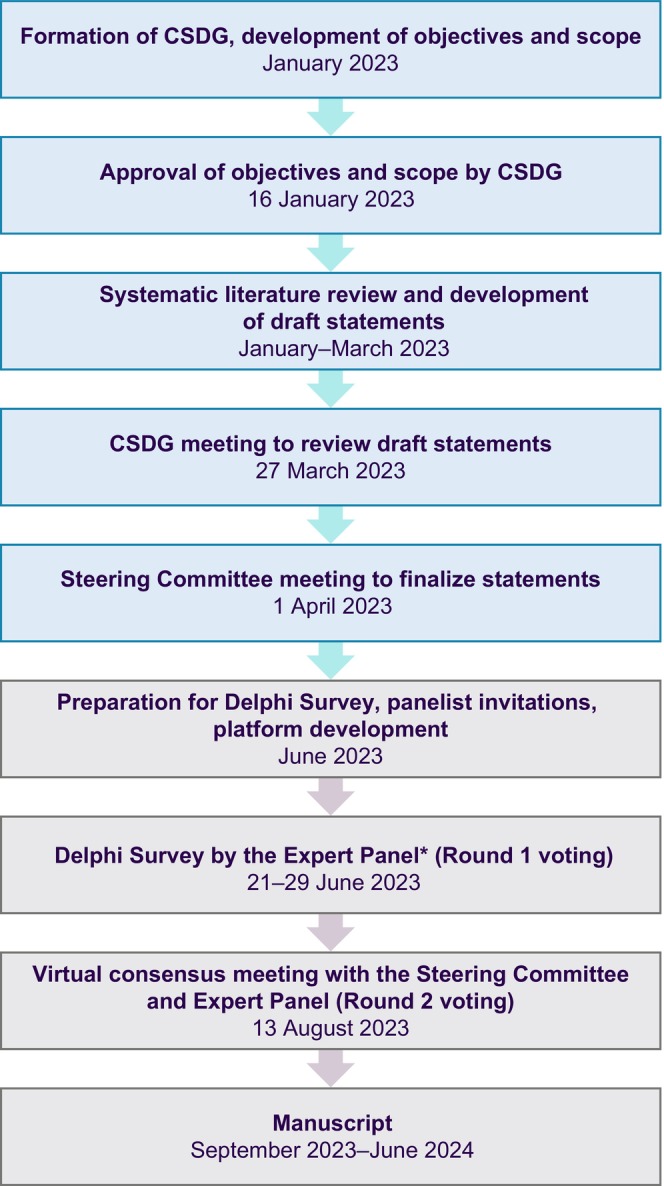
Overview of the consensus development process. *Via Survey Monkey platform. Abbreviation: CSDG, Consensus Statement Development Group.

A Steering Committee (SC) of eight globally recognized GPP experts from the APAC region guided this study. The Consensus Statement Development Group (CSDG), comprising three co‐chairs within the SC, substantiated the findings of a systematic literature review (SLR) and directed statement development. A total of 140 statements were developed based on the collective expertise of the SC and the SLR, which adhered to the PRISMA guidelines. Comprehensive information regarding the SLR methodology and the demographic profile of the SC is available in the Supporting Information. The statements encompassed a wide spectrum of clinical, laboratory, histologic features, and treatment strategies crucial for formulating recommendations for the management of GPP. To ensure a rigorous and iterative consensus‐building process, these statements underwent evaluation in a two‐round Delphi study (Figure 1).

### 2.1 Expert Panel selection

Twelve additional GPP experts from the APAC region, identified by the SC based on their clinical expertise on GPP, were invited via email to participate in two consensus rounds. All agreed to participate. Together with the eight SC members, a panel of 20 GPP experts contributed actively to this consensus study.

### 2.2 Delphi process

During the initial Delphi round, voting took place through Survey Monkey, with consensus defined as ≥80% agreement on a given statement. The results of this voting, along with insights from the panelists, underwent thorough review by the SC. Statements achieving ≥80% consensus were retained without revision unless deemed necessary by the CSDG. Statements falling below the agreement threshold were revised or removed after in‐depth deliberation. The revised statements, along with the new statements derived from the feedback in the Delphi survey (Round 1), were subjected to discussion and voting in a virtual consensus meeting during the second round (Round 2).

## 3 RESULTS

### 3.1 Systematic literature review

The SLR identified 4091 articles from 1980 to January 19, 2023 (Table S1), of which 768 were eligible for full‐text review after title and abstract screening. A total of 361 articles were included following full‐text review (Figure S1).

### 3.2 Demographics of Delphi panelists

Our Expert Panel consists of 20 GPP experts from Australia, China, Japan, Korea, Malaysia, Singapore, Taiwan, Thailand, the Philippines, and Vietnam (Table S2). Among the panelists, 20% worked in an academic hospital setting, 35% in a public hospital, 10% in private setting, and 20% in both academic and public hospitals. The panelists managed an average of 39 GPP cases over the last 5 years and the majority (75%) had over 20 years of clinical experience. The mean age of panelists was 53.4 (range 40–67) years and 55% were male. The first Delphi consensus survey (Round 1) was completed by 12 experts, while 20 participated in the virtual consensus meeting (Round 2).

### 3.3 Modified Delphi study findings

**FIGURE 2:**
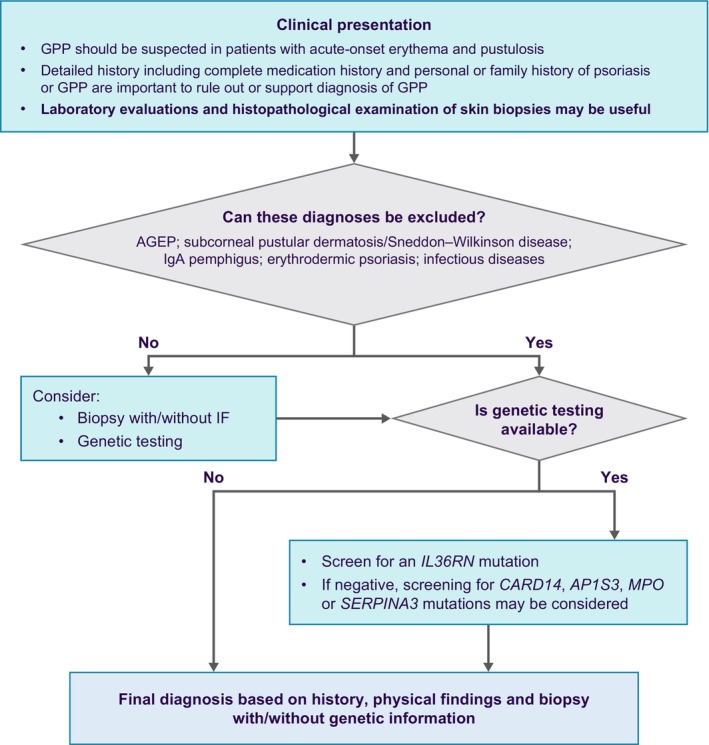
Diagnostic algorithm for patients with GPP. Abbreviations: AGEP, acute generalized exanthematous pustulosis; AP1S3, adaptor related protein complex 1 subunit sigma 3; CARD14, caspase recruitment domain family member 14; GPP, generalized pustular psoriasis; IF, immunofluorescence; IgA, immunoglobulin A; MPO, myeloperoxidase; SERPINA3, serpin family A member 3.

**FIGURE 3:**
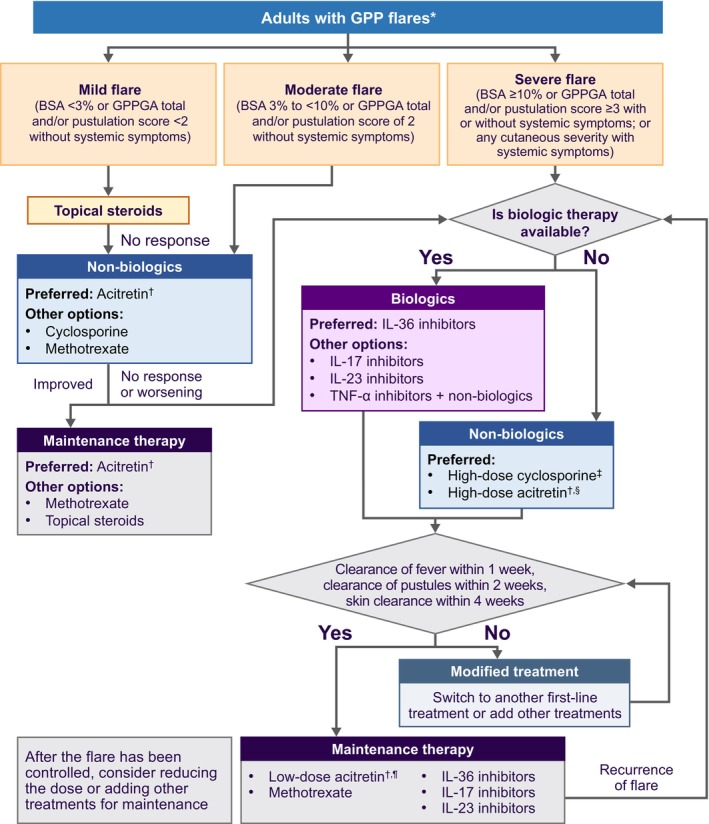
Treatment algorithm for the management of adults with GPP flares. *The severity classification of GPP flares is intended as a guide only and should not be used to limit treatment access. Treatment choice should follow local guidelines. ^†^Etretinate if acitretin is not available. ^‡^3.5–5 mg/kg. ^§^0.5–1 mg/kg. ^¶^0.125–0.5 mg/kg. Abbreviations: BSA, body surface area; GPP, generalized pustular psoriasis; GPPGA, Generalized Pustular Psoriasis Physician Global Assessment; IL, interleukin; TNF, tumor necrosis factor.

In Round 1, 80 statements (57% of 140) achieved consensus. Of the 60 statements that failed to secure consensus, 57% (34/60) were declared “no consensus” (Tables 1 and 2). During Round 2, 35 statements (four and 26 statements that reached and did not reached consensus during Round 1, respectively, and five new statements developed based on the panelists' feedback) were presented for discussion (Tables S3 and S4). These revised statements underwent further refinement with valuable input from the experts. During the deliberations, consensus was reached to exclude six statements from the subsequent re‐voting session, while nine new statements were proposed and added for voting (Table S4). Following the re‐voting session, a total of 30 statements, including the four statements reaching consensus in Round 1, ultimately achieved consensus. This brought the cumulative number of statements in consensus to 106, which served as the foundation for the development of both diagnostic and treatment algorithms for GPP (Figures 2 and 3).

**TABLE 1:** Statements that achieved consensus

**1. Definition and clinical course**	**Consensus**	
**GPP definition and terminology**	**Round 1** ^a^ **(%)**	**Round 2** ^b^ **(%)**
1.1A. GPP is defined as primary, sterile, macroscopically visible, extensive skin pustules that can be associated with systemic inflammation	100%	NA
1.1B. GPP mainly affects non‐acral regions but acral regions may be affected	92%	NA
1.1C. GPP is phenotypically, genetically, and histopathologically distinct from plaque psoriasis	92%	NA
**Epidemiology of GPP**	**Round 1** ^a^ **(%)**	**Round 2** ^b^ **(%)**
1.2A. GPP is a rare and severe form of pustular psoriasis	83%	NA
1.2B. GPP is more prevalent in females	83%	NA
1.2C. GPP is more prevalent in Asia; however, the prevalence may vary across the region	83%	NA
**Classification of GPP**	**Round 1** ^a^ **(%)**	**Round 2** ^b^ **(%)**
1.3A. GPP can present as acute form with widespread pustular eruption or subacute variant with annular phenotype, with tendency of transforming from one form to another	100%	NA
1.3B. GPP may either be relapsing or persistent, with relapsing form being more common	92%	NA
1.3C. GPP can be classified based on disease onset into pediatric and adult‐onset GPP	83%	NA
1.3D. Patients with GPP may or may not have associated plaque psoriasis	92%^c^	100%
1.3E. Patients with GPP may or may not have *IL36RN* mutations	100%	NA
1.3F. Patients with GPP may or may not have *CARD14* mutations	92%	NA
**Signs and symptoms**	**Round 1** ^a^ **(%)**	**Round 2** ^b^ **(%)**
1.4A. Pustules, erythema, burning, pain, and discomfort are the common signs and symptoms seen in patients with GPP	100%	NA
1.4B. The common systemic symptoms include fever, chills, malaise, and fatigue	100%	NA
1.4C. Mucocutaneous symptoms such as geographic tongue or fissured tongue may also occur in patients with GPP	100%	NA
**Flare definition and clinical course**	**Round 1** ^a^ **(%)**	**Round 2** ^b^ **(%)**
1.5A. Flares are a hallmark of GPP and can be defined as the sudden eruption of new sterile pustules with or without systemic symptoms	100%	NA
1.5B. GPP flares that affect over 10% of body surface area can be defined as severe GPP	92%	NA
1.5C. GPP flares that affect at least 10% of the body surface area can be defined as severe GPP	NA^d^	100%
1.5D. GPP flares that affect less than 3% of the body surface area with concomitant systemic symptoms can be defined as severe GPP	NA^d^	90%
1.5E. GPPGA total score of at least 3 can be defined as severe GPP	92%	NA
1.5F. GPPGA pustulation score of at least 3 can be defined as severe GPP	83%	NA
1.5G. GPP flares that affect <3% of body surface area without concomitant systemic symptoms can be defined as mild GPP	NA^d^	100%
1.5H. GPPGA total score <2 can be defined as mild GPP	NA^d^	94%
1.5I. GPPGA pustulation score <2 can be defined as mild GPP	NA^d^	100%
1.5 J. Patients with GPP may have clear skin between flares, except in the setting of concomitant plaque psoriasis	42%^e^	100%
1.5 K. Patients with GPP may have residual disease such as erythema with pustules between flares	75%^e^	84%
1.5 L. Most GPP flares last 2–5 weeks	83%	NA
**Triggers and risk factors for GPP**	**Round 1** ^a^ **(%)**	**Round 2** ^b^ **(%)**
1.6A. Systemic steroids, particularly during tapering or withdrawing, may trigger GPP flares	92%	NA
1.6B. Stress may trigger GPP flares	100%	NA
1.6C. Infections may trigger GPP flares	100%	NA
1.6D. Menstruation may trigger GPP flares	92%	NA
1.6E. Pregnancy may trigger GPP flares	100%	NA
1.6F. Vaccination may trigger GPP flares.	100%	NA
Complications, comorbidities, and prognosis	Round 1^a^ (%)	Round 2^b^ (%)
1.7A. Plaque psoriasis, psoriatic arthritis, depression, anxiety, hypertension, diabetes, and hyperlipidemia are the common comorbidities of GPP among Asian patients	83%	NA
1.7B. Older patients with GPP may have poorer prognosis due to comorbidities	92%	NA
1.7C. GPP is a potentially life‐threatening condition	100%	NA
1.7D. GPP has a substantial impact on patients' quality of life	100%	NA
**2. Diagnosis of GPP**		
**Diagnostic criteria**	**Round 1** ^a^ **(%)**	**Round 2** ^b^ **(%)**
2.1A. GPP should be diagnosed in patients presenting with primary, sterile, macroscopically visible, extensive skin pustules with or without systemic inflammation and with or without plaque psoriasis	83%	NA
**Medical and family history**	**Round 1** ^a^ **(%)**	**Round 2** ^b^ **(%)**
2.2A. A positive family history of psoriasis or GPP supports diagnosis of GPP	83%	NA
**Histological features of GPP**	**Round 1** ^a^ **(%)**	**Round 2** ^b^ **(%)**
2.3A. A skin biopsy is not mandatory but may be necessary to rule out differential diagnoses	83%	NA
2.3B. Key histological features of GPP include neutrophil infiltration, Kogoj's spongiform pustules, and Munro's microabscesses	92%	NA
**Genetic screening**	**Round 1** ^a^ **(%)**	**Round 2** ^b^ **(%)**
2.4A. Implementation of genetic screening may offer the opportunity to identify GPP early, detect certain forms of GPP, personalize treatment strategies, and predict treatment outcomes	100%	NA
**Differential diagnoses**	**Round 1** ^a^ **(%)**	**Round 2** ^b^ **(%)**
2.5A. A diagnosis of GPP requires careful assessment and ruling out conditions with similar skin symptoms, such as AGEP, other forms of psoriasis, autoimmune disorders, and infections	100%	NA
2.5B. AGEP is the most important differential diagnosis of GPP and should be actively ruled out	100%	NA
**3. Treatment outcomes, goals, and monitoring measures for GPP**		
**Short‐term/flare‐phase treatment goals**	**Round 1** ^a^ **(%)**	**Round 2** ^b^ **(%)**
3.1A. The immediate therapeutic goal should be rapid resolution of cutaneous and systemic signs and symptoms of GPP flares	100%	NA
3.1B. Treatment goal should be clearance of pustules and resolution of fever as soon as possible, preferably within 1 week, with skin clearance within 4 weeks	75%^e^	100%
**Long‐term treatment goals**	**Round 1** ^a^ **(%)**	**Round 2** ^b^ **(%)**
3.2A. One of the key treatment goals in patients with GPP is maintenance of response and prevention of flares	100%	NA
3.2B. Skin symptoms should be monitored using GPP‐specific measures to identify changes in disease severity and treatment response	100%	NA
3.2C. Choice of treatment for GPP is based on the disease severity and comorbidities	100%	NA
3.2D. Due to the substantial emotional burden of GPP beyond the physical discomfort of skin lesions, improving patients' quality of life through effective treatments is an important treatment goal	75%^e^	100%
**Assessment tools for measuring disease severity and treatment response**	**Round 1** ^a^ **(%)**	**Round 2** ^b^ **(%)**
3.3A. Laboratory tests indicative of systemic inflammation should be considered for assessing the disease severity and the risk of potential complications associated with GPP	100%	NA
3.3B. Cardiopulmonary comorbidities should be assessed for patients with GPP using appropriate imaging and laboratory tests	92%	NA
3.3C. GPPGA should be routinely used to assess disease severity and treatment response in clinical practice	92%	NA
3.3D. DLQI should be used in routine clinical practice to assess treatment response and patients' quality of life	83%	NA
3.3E. Pain VAS should be used in routine clinical practice to assess treatment response and patients' quality of life	83%	NA
**4. Optimal management strategies and clinical practices**		
**Treatment strategies**	**Round 1** ^a^ **(%)**	**Round 2** ^b^ **(%)**
4.1A. Treatments with rapid onset of action are essential for patients with GPP flares	100%	NA
4.1B. Currently, biologics are the preferred treatment of choice when managing acute flares, if accessible	75%^e^	100%
4.1C. Maintenance treatment is generally needed to control residual lesions (including ACH) and prevent new/recurrent flares	100%	NA
**Systemic treatment for flare and maintenance phase**		
**Flare phase: Preferred therapy**	**Round 1** ^a^ **(%)**	**Round 2** ^b^ **(%)**
4.1D. IL‐36 inhibitors are recommended as first‐line treatment to manage acute flares	92%	NA
4.1E. High‐dose acitretin is recommended as first‐line treatment to manage acute flares when biologics are not available/accessible	55%^e^	84%
4.1F. High‐dose cyclosporine is recommended as first‐line treatment to manage severe acute flares when biologics are not available/accessible	75%^e^	95%
4.1G. IL‐17 inhibitors can be considered for managing acute flares if other preferred therapies are not accessible	60%^e^	89%
4.1H. High‐dose acitretin can be considered as second‐line treatment to manage acute flares	58%^e^	88%
4.1I. What is the preferred or recommended first‐line treatment for mild GPP? Acitretin	NA^d^	89%
**Maintenance phase: Preferred therapy**	**Round 1** ^a^ **(%)**	**Round 2** ^b^ **(%)**
4.1J. Low‐dose acitretin is the recommended treatment for maintenance phase	83%	NA
4.1K. Methotrexate is the recommended treatment for maintenance phase	75%^e^	95%
4.1L. IL‐36 inhibitors can be used for maintenance phase	60%^e^, ^f^	83%^f^
4.1M. IL‐17 inhibitors can be used for maintenance phase	79%^e^, ^f^	100%
4.1N. IL‐23 inhibitors can be used for maintenance phase	50%^e^, ^f^	94%^f^
4.1O. What is the preferred or recommended maintenance treatment for mild GPP? Acitretin	NA^d^	85%
**Non‐biologic treatments for the management of GPP**	**Round 1** ^a^ **(%)**	**Round 2** ^b^ **(%)**
4.2A. Cyclosporine should not be used for long‐term maintenance beyond 1–2 years	92%	NA
4.2B. Methotrexate can be considered for long‐term treatment	92%	NA
4.2C. If patients cannot tolerate high‐dose non‐biologic treatment for an extended period of time after the acute flare has been controlled, consider reducing the dose and adding other treatments for maintenance	100%	NA
4.2D. Withdrawal of systemic treatments can result in relapse	100%	NA
4.2E. Systemic antibiotics should be considered only if there is a clear indication of infections or if infections cannot be ruled out during the acute phase	83%	NA
4.2F. In general, systemic corticosteroids are not recommended as maintenance therapy in patients with GPP	92%	NA
4.2G. Phototherapy is not recommended for the management of acute flares	92%	NA
4.2H. If the patient's condition improves within 2–4 weeks of starting systemic treatments in the acute phase (pustule improvement, no appearance of new lesions), the dose of non‐biologic treatment can be tapered gradually according to clinical response. Abrupt and/or early tapering may result in flares and suboptimal disease control	75%^e^	95%^f^
**Biologic treatments for the management of GPP**	**Round 1** ^a^ **(%)**	**Round 2** ^b^ **(%)**
4.3A. Spesolimab is the preferred biologic treatment for the management of acute flares, as it results in rapid improvements in skin symptoms following a single dose in patients with or without *IL36RN* mutations	100%	NA
4.3B. Patients with *IL36RN* mutations respond to spesolimab faster.	88%^f^	NA
4.3C. IL‐17 inhibitors and IL‐23 inhibitors can be considered for the management of acute flares	90%^f^	NA
4.3D. TNF‐α inhibitors may require concomitant treatment with non‐biologics	90%^f^	NA
4.3E. TNF‐α inhibitors are not recommended for patients with active or latent tuberculosis (TB); they can be used in treated TB or 1 month after commencement of treatment for latent TB	83%^c^	100%
4.3F. As with systemic medications, due to associated side effects and the possibility of rebound, patients receiving biologics should be carefully monitored	92%	NA
4.3G. Treatment should be decided based on the patient's condition and availability of biologics in individual countries	100%	NA
**Management of childhood GPP**	**Round 1** ^a^ **(%)**	**Round 2** ^b^ **(%)**
4.4A. Specific support and care are needed from specialists when managing pediatric patients with GPP	100%	NA
4.4B. Acitretin can be used for the management of GPP in pediatric patients when biologics are not available/accessible	70%^e^	100%^f^
4.4C. Acitretin is recommended for the management of acute GPP flares in children	NA^d^	94%
4.4D. Cyclosporine is recommended for the management of GPP in pediatric patients	91%^f^	NA
4.4E. Methotrexate is recommended for the management of GPP in pediatric patients	91%^f^	NA
4.4F. IL‐17 inhibitors are recommended for the management of GPP in pediatric patients	100%^f^	NA
4.4G. TNF‐α inhibitors are recommended for the management of GPP in pediatric patients	100%^f^	NA
4.4H. IL‐36 inhibitors may be considered for the management of acute GPP flares in children who failed the standard treatments	NA^d^	100%
**Management of GPP in pregnancy**	**Round 1** ^a^ **(%)**	**Round 2** ^b^ **(%)**
4.5A. Close systemic monitoring and sufficient supportive treatment during pregnancy are required to control GPP	100%	NA
4.5B. Cyclosporine is recommended for the management of GPP in pregnant patients	83%	NA
4.5C. Low‐dose systemic corticosteroids may be considered for the management of GPP in pregnant patients if other treatment options fail/are not available	67%^e^	83%^f^
4.5D. Biologics should be carefully considered when treating GPP in pregnant patients based on risk–benefit profile for individual patients	100%	NA
4.5E. TNF‐α inhibitors^g^ should be considered carefully when treating GPP in pregnant patients based on the risk–benefit profile for individual patients	60%^e^, ^f^	100%^f^
4.5F. Dermatologists should work closely with OB‐GYNs to prevent any negative outcome	92%^c^	100%
4.5G. Dermatologists should work closely with pediatricians and caregivers following delivery to prevent negative outcomes to the mother and child	92%	NA
**Treatment strategies for ACH**	**Round 1** ^a^ **(%)**	**Round 2** ^b^ **(%)**
4.6A. The same treatments that are used for the maintenance of GPP (i.e., methotrexate, cyclosporine, acitretin) are recommended when managing ACH	92%	NA
4.6B. Biologic agents are especially recommended for recalcitrant ACH	92%	NA
**Holistic management for patients with GPP**	**Round 1** ^a^ **(%)**	**Round 2** ^b^ **(%)**
4.7A. Beyond pharmacological intervention, lifestyle modifications are helpful to ensure optimal treatment outcomes	100%	NA
4.7B. Patients should avoid smoking and trauma, and manage stress	100%^c^	85%
4.7C. A multidisciplinary treatment approach led by dermatologists with input from other specialties, including ICU, where appropriate, is recommended	100%	NA
4.7D. Psychological follow‐up and genetic counseling for patients can be considered	100%	NA

Abbreviations: ACH, acrodermatitis continua of Hallopeau; AGEP, acute generalized exanthematous pustulosis; CARD14, caspase recruitment domain family member 14; DLQI, Dermatology Life Quality Index; GPP, generalized pustular psoriasis; GPPGA, Generalized Pustular Psoriasis Physician Global Assessment; ICU, intensive care unit; IL, interleukin; OB‐GYN, obstetrician‐gynecologist; TB, tuberculosis; TNF, tumor necrosis factor; VAS, Visual Analogue Scale.

^a^ Round 1: Consensus achieved during the online Delphi survey (involving Delphi Expert Panel).

^b^ Round 2: Consensus achieved during the virtual consensus meeting (involving both Steering Committee and Delphi Expert Panel).

^c^ Statements that achieved consensus during Delphi survey (Round 1) but were improved on based on the Delphi panelists' feedback. Refer to Supporting Information Table S3 for the original version of the statement that was shared during Delphi survey (Round 1).

^d^ New statements that were included following Delphi survey (Round 1) and achieved consensus during the virtual consensus meeting (Round 2).

^e^ Statements that did not achieve consensus during Delphi survey (Round 1) and were revised based on the Delphi Panelists' feedback and were voted/achieved consensus during the virtual consensus meeting (Round 2). Refer to Supporting Information Table S3 for the original version of the statement that was shared during Delphi survey (Round 1).

^f^ Number of experts who selected ‘I don't have relevant experience’ is excluded when calculating the consensus.

^g^ Certolizumab pegol is the preferred TNF‐α inhibitor.

**TABLE 2:** Statements that did not reach consensus.

**1. Definition and clinical course**	**Consensus**	
**Epidemiology of GPP**	**Round 1** ^a^ **(%)**	**Round 2** ^b^ **(%)**
GPP onset is less common in children than in adults	67%	NA
GPP is less common in children	NA	74%
**Flare definition and clinical course**	**Round 1** ^a^ **(%)**	**Round 2** ^b^ **(%)**
GPP flares that affect over 5% of body surface area can be defined as severe GPP	25%	NA
GPP flares that affect over 25% of body surface area can be defined as severe GPP	58%	NA
GPP flares are self‐limiting	33%	NA
**Complications, comorbidities, and prognosis**	**Round 1** ^a^ **(%)**	**Round 2** ^b^ **(%)**
Renal failure and liver diseases are the common complications of GPP observed in Asian patients	33%	NA
Obesity is a common comorbidity among Asian patients with GPP	NA	28%
**2. Diagnosis of GPP**		
**Medical and family history**	**Round 1** ^a^ **(%)**	**Round 2** ^b^ **(%)**
A history of concurrent or previous plaque psoriasis supports the diagnosis of GPP	58%	NA
A family history of acrodermatitis continua of Hallopeau supports the diagnosis of GPP	67%	NA
GPP is distinct from GPP with plaque psoriasis	NA	74%
**Histological features of GPP**	**Round 1** ^a^ **(%)**	**Round 2** ^b^ **(%)**
A skin biopsy is mandatory for the diagnosis of GPP	17%	NA
**Genetic screening**	**Round 1** ^a^ **(%)**	**Round 2** ^b^ **(%)**
Genetic screening is recommended for diagnosis of GPP	50%	NA
Genetic screening is mandatory for prognosis of GPP	25%	NA
**3. Treatment outcomes, goals, and monitoring measures for GPP**		
**Short‐term/flare‐phase treatment goals**	**Round 1** ^a^ **(%)**	**Round 2** ^b^ **(%)**
Treatment goal should be pustular clearance and resolution of fever within 1 week	42%	NA
Treatment goal should be pustular clearance and resolution of fever within 2 weeks	25%	NA
Treatment goal should be clearance of pustules and resolution of fever as soon as possible, preferably within 2 weeks, with skin clearance within 4 weeks	NA	32%
**Assessment tools for measuring disease severity and treatment response**	**Round 1** ^a^ **(%)**	**Round 2** ^b^ **(%)**
GPPASI should be routinely used to assess disease severity and treatment response in clinical practice	67%	NA
CGI should be routinely used to assess disease severity and treatment response in clinical practice	42%	NA
**4. Optimal management strategies and clinical practices**		
**Systemic treatment for flare and maintenance phase**		
**Flare phase**	**Round 1** ^a^ **(%)**	**Round 2** ^b^ **(%)**
Etretinate has similar efficacy to acitretin and can be used as an alternative to acitretin as first‐line treatment to manage acute flares	38%^c^	NA
Methotrexate is recommended as first‐line treatment to manage acute flares	25%	NA
IL‐23 inhibitors are recommended as first‐line treatment to manage acute flares	33%^c^	NA
TNF‐α inhibitors are recommended as first‐line treatment to manage acute flares	40%^c^	NA
IL‐12/IL‐23 inhibitors are recommended as first‐line treatment to manage acute flares	13%^c^	NA
IL‐1 inhibitors are recommended as first‐line treatment to manage acute flares	0%^c^	NA
IL‐12/IL‐23 inhibitors are recommended as second‐line treatment to manage acute flares	38%^c^	NA
IL‐1 inhibitors are recommended as second‐line treatment to manage acute flares	40%^c^	NA
High‐dose cyclosporine is recommended as second‐line treatment to manage acute flares	58%^c^	NA
Methotrexate is recommended as second‐line treatment to manage acute flares	67%	NA
IL‐36 inhibitors are recommended as second‐line treatment to manage acute flares	58%	NA
IL‐17 inhibitors are recommended as second‐line treatment to manage acute flares	60%^c^	NA
IL‐23 inhibitors are recommended as second‐line treatment to manage acute flares	67%^c^	NA
TNF‐α inhibitors are recommended as second‐line treatment to manage acute flares	60%^c^	NA
What is the preferred or recommended first‐line treatment for mild GPP?	NA	
Cyclosporine		67%
Methotrexate		61%
Topical steroids		61%
Other treatments		17%
What is the preferred or recommended maintenance treatment for mild GPP?	NA	
Cyclosporine		30%
Methotrexate		70%
Topical steroids		55%
Other treatments		20%
**Maintenance phase: Preferred therapy**	**Round 1** ^a^ **(%)**	**Round 2** ^b^ **(%)**
Low‐dose cyclosporine is the recommended treatment for maintenance phase	67%	NA
Low‐dose cyclosporine can be used for maintenance phase	NA	70%
IL‐1 inhibitors are the recommended treatment for maintenance phase	25%^c^	NA
IL‐12/IL‐23 inhibitors are the recommended treatment for maintenance phase	14%^c^	NA
TNF‐α inhibitors are the recommended treatment for maintenance phase	56%^c^	NA
TNF‐α inhibitors can be used for maintenance phase	NA	69%
**Non‐biologic treatments for the management of GPP**	**Round 1** ^a^ **(%)**	**Round 2** ^b^ **(%)**
In general, systemic corticosteroids are not recommended for the management of acute GPP flares	58%	NA
Non‐pharmacological treatments, such as GMA and IVIG, can be considered based on their availability in individual countries	50%^c^	NA
**Management of childhood GPP**	**Round 1** ^a^ **(%)**	**Round 2** ^b^ **(%)**
IL‐36 inhibitors are recommended for the management of GPP in pediatric patients	67%^c^	NA
IL‐23 inhibitors are recommended for the management of GPP in pediatric patients	50%^c^	NA
IL‐12/IL‐23 inhibitors are recommended for the management of GPP in pediatric patients	43%^c^	NA
IL‐1 inhibitors are recommended for the management of GPP in pediatric patients	33%^c^	NA
Spesolimab is recommended for the management of acute GPP flares in children	NA	27%
Spesolimab may be considered for the management of acute GPP flares in children	NA	79%
**Management of GPP in pregnancy**	**Round 1** ^a^ **(%)**	**Round 2** ^b^ **(%)**
IL‐36 inhibitors are recommended for the management of GPP in pregnant patients	30%^c^	NA
IL‐17 inhibitors are recommended for the management of GPP in pregnant patients	22%^c^	NA
IL‐23 inhibitors are recommended for the management of GPP in pregnant patients	0%^c^	NA
IL‐12/IL‐23 inhibitors are recommended for the management of GPP in pregnant patients	0%^c^	NA
IL‐1 inhibitors are recommended for the management of GPP in pregnant patients	17%^c^	NA

Abbreviations: CGI, Clinical Global Impression; GMA, adsorptive granulocyte and monocyte apheresis; GPP, generalized pustular psoriasis; GPPASI, Generalized Pustular Psoriasis Area and Severity Index; IL, interleukin; IVIG, intravenous immunoglobulin; TNF, tumor necrosis factor.

^a^ Round 1 voting during the online Delphi survey (involving Delphi Expert Panel).

^b^ Round 2 voting during the virtual consensus meeting (involving both Steering Committee and Delphi Expert Panel).

^c^ Number of experts who selected ‘I don't have relevant experience’ was excluded when calculating the consensus.

Consensus was reached that GPP is defined as primary, sterile, macroscopically visible, extensive skin pustules, potentially associated with systemic inflammation (Table 1). It mainly affects non‐acral regions but acral regions may also be affected. GPP was acknowledged as phenotypically, genetically, and histopathologically distinct from plaque psoriasis, with varying prevalence across Asia. While consensus was not reached on GPP onset being less common in children than in adults, agreement existed on its relapsing nature and common clinical manifestations (Tables 1 and 2). Consensus was also established for diagnosing GPP in patients presenting with primary, sterile, macroscopically visible, extensive skin pustules with or without systemic inflammation, and with or without plaque psoriasis. A positive family history of psoriasis or GPP supports the diagnosis of GPP. Key histological features of GPP include neutrophil infiltration, Kogoj's spongiform pustules, and Munro's microabscesses. However, a skin biopsy was deemed not mandatory for diagnosis but may be necessary to rule out other neutrophilic eruptions such as acute generalized exanthematous pustulosis (AGEP). Genetic testing is not obligatory but recommended if available. Detection of genetic mutations, such as *IL36RN*, may identify patients who may need more vigilant monitoring and should be prioritized for targeted therapy. Consensus was successfully reached on both the definition of a flare and the classification of flare severity.

The experts agreed that the immediate therapeutic goal should be rapid resolution of cutaneous and systemic signs and symptoms of GPP flares. During a flare, the treatment goal is defined by the resolution of fever as soon as possible, preferably within 1 week, clearance of pustules within 2 weeks, and skin clearance within 4 weeks (Figure 3).

Regarding long‐term treatment goals, the experts concurred that the maintenance of response and prevention of flares are paramount. Recognizing the substantial emotional burden of GPP beyond the physical discomfort of skin lesions, improving patients' quality of life (QoL) through effective treatments is an important treatment goal.

The experts unanimously advocate regular laboratory tests to assess disease severity and monitor the risk of potential complications during a flare. Generalized Pustular Psoriasis Physician Global Assessment (GPPGA), a validated GPP‐specific severity assessment tool,^32^ should be routinely used to assess disease severity and treatment response in clinical practice, while Dermatology Life Quality Index (DLQI) and Pain Visual Analogue Scale (Pain‐VAS) are useful tools to assess the impact of GPP on patients' QoL.

The experts agreed that treatments with a rapid onset of action are essential for patients with GPP flares, while maintenance treatment is generally needed to control chronic symptoms and prevent new/recurrent flares. An expert‐drafted treatment algorithm for adult GPP serves as a guideline for effective management (Figure 3). For adults experiencing flares, the severity of the flare determines the management approach. Considering the lack of an established classification, the panel proposed a severity classification of GPP flares (Table 3). This classification is intended as a guide only and should not be used to limit treatment access. Treatment choice should follow local guidelines.

**TABLE 3:** Definition and severity classification of GPP flares.^a^

Definition	
Flare is defined as the sudden eruption of new sterile pustules with or without systemic symptoms	
Severity	
Mild	BSA <3% or GPPGA total and/or pustulation score <2 without systemic symptoms
Moderate	BSA 3% to <10% or GPPGA total and/or pustulation score of 2 without systemic symptoms
Severe	BSA ≥10% or GPPGA total and/or pustulation score ≥3 with or without systemic symptoms, or any cutaneous severity with systemic symptoms

Abbreviations: BSA, body surface area; GPP, generalized pustular psoriasis; GPPGA, Generalized Pustular Psoriasis Physician Global Assessment.

^a^ The severity classification of GPP flares is intended as a guide only and should not be used to limit treatment access. Treatment choice should follow local guidelines.

Mild flares (body surface area [BSA] <3% or GPPGA total and/or pustulation score <2 without systemic symptoms) may be initially addressed with topical steroids as first‐line treatment. In cases of inadequate response, non‐biologic treatments should be considered, with acitretin as the preferred option, along with alternatives like cyclosporine and methotrexate. Improvement allows for transitioning to maintenance treatment, while lack of response or flare worsening should prompt consideration of biologics.

Moderate flares (BSA 3% to <10% or GPPGA total and/or pustulation score of 2 without systemic symptoms) are best managed with non‐biologics, with acitretin as the preferred choice and cyclosporine and methotrexate as alternative treatment options. Similar to mild flares, if improvement occurs, maintenance treatment is initiated; otherwise, biologics should be considered. Maintenance treatment strategies for mild and moderate flares are consistent, with acitretin as the preferred option and other choices including methotrexate and topical steroids.

Severe flares (BSA ≥10% or GPPGA total and/or pustulation score ≥3 with or without systemic symptoms, or any cutaneous severity with systemic symptoms) necessitate highly efficacious and fast‐acting biologics, with IL‐36 inhibitors preferred as the first‐line treatment, if available. Spesolimab is advocated as the first‐line treatment as it is the only biologic with robust evidence of its efficacy and safety in rapidly cooling down the inflammation of GPP flares. Alternatives encompass IL‐17 inhibitors, IL‐23 inhibitors, and TNF‐α inhibitors as monotherapy or in combination with non‐biologics. TNF‐α inhibitors are cautioned against for patients with active or latent tuberculosis. In the absence of biologic therapy, high‐dose acitretin (0.5–1 mg/kg) or high‐dose cyclosporine (3.5–5 mg/kg) is recommended. Gradual tapering of non‐biologic treatment is advised on improvement, with abrupt or early tapering posing risks of relapse and suboptimal disease control.

Following treatment, if fever resolution is achieved within 1 week, clearance of pustules within 2 weeks, and skin clearance within 4 weeks, treatment should be switched to maintenance therapy, with recommended options being low‐dose acitretin (0.125–0.5 mg/kg), methotrexate, IL‐36 inhibitors, IL‐17 inhibitors, or IL‐23 inhibitors. Failure to achieve treatment goals should prompt treatment modification before transitioning to maintenance therapy. Given the potential side effects and rebound effects with biologics, vigilant patient monitoring is stressed. Treatment decisions should align with individual patient conditions and the availability of biologics in specific countries.

**FIGURE 4:**
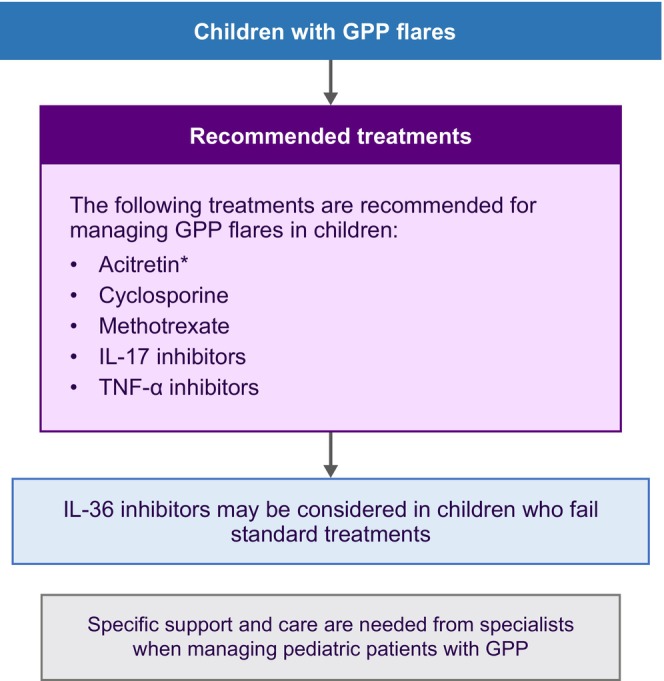
Treatment algorithm for the management of childhood GPP. *Etretinate if acitretin is not available. Abbreviations: GPP, generalized pustular psoriasis; IL, interleukin; TNF, tumor necrosis factor.

The consensus among experts underscores the need for specialized support and care when managing pediatric patients with GPP. The treatment algorithm for childhood GPP illustrated in Figure 4 provides a comprehensive guide based on expert consensus. Acitretin, cyclosporine, methotrexate, IL‐17 inhibitors, and TNF‐α inhibitors are the recommended treatment options for childhood GPP. IL‐36 inhibitors are deemed appropriate for children who failed standard treatments, as subcutaneous spesolimab was recently approved in the USA for the treatment of GPP in adults and pediatric patients 12 years of age and older and weighing ≥40 kg, and received expanded approval in China for the reduction of occurrence of GPP flares in adults and adolescents from 12 years of age with a body weight ≥40 kg.^33^ The recommendation is to evaluate the potential benefits and risks on an individual basis, emphasizing a careful consideration of the risk–benefit profile.

**FIGURE 5:**
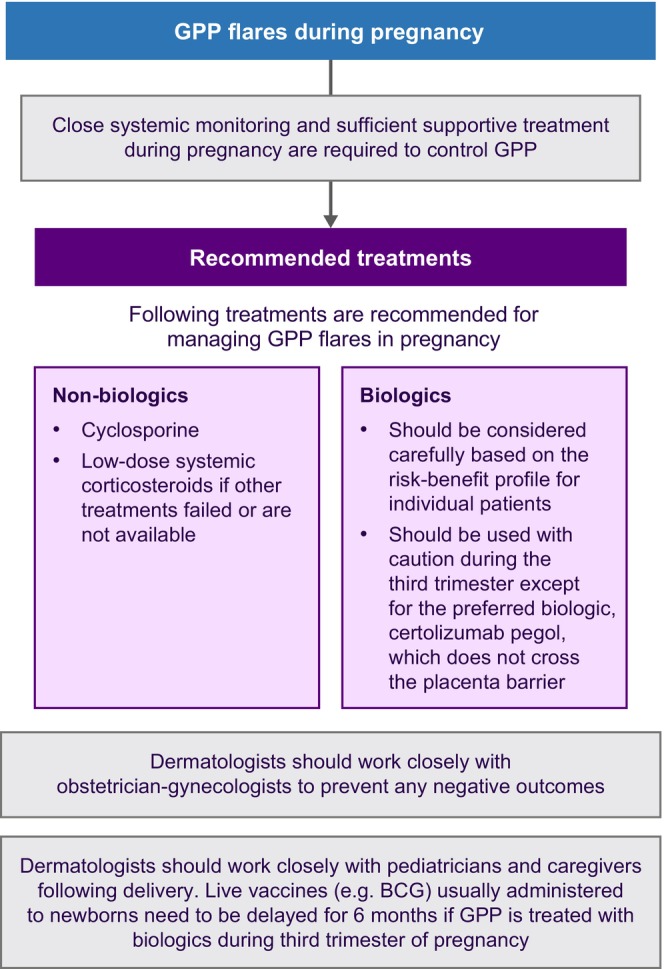
Treatment algorithm for the management of GPP in pregnancy. Abbreviations: BCG, Bacillus Calmette–Guérin; GPP, generalized pustular psoriasis.

In the context of pregnancy, the experts advocate for close systemic monitoring and supportive treatment to effectively manage GPP. The consensus‐driven treatment algorithm in Figure 5 suggests cyclosporine and low‐dose systemic corticosteroids as recommended treatments during pregnancy. Among biologics, the TNF‐α inhibitor certolizumab pegol is preferred as it does not cross the placenta.^34^, ^35^ Dermatologists should work closely with the obstetrician/gynecologists to prevent any negative outcomes; they should also work closely with pediatricians/neonatologists and caregivers following delivery. Live vaccines (e.g., BCG) usually administered to newborns should be delayed for 6 months if GPP is treated with biologics during the third trimester of pregnancy.

Beyond pharmacological interventions, lifestyle modifications are deemed beneficial for optimal treatment outcomes. Patients should be encouraged to avoid smoking and manage their stress levels. A multidisciplinary treatment approach led by dermatologists with input from other specialties, including intensive care specialists, where appropriate, is recommended when managing patients with GPP. If accessible, psychological follow‐up and genetic counseling for patients may be considered.

Access, reimbursement, and cost are the major factors limiting the use of biologics in the APAC region. Approval and availability of biologics in the APAC region are summarized in Table 4. Efforts should be directed toward improving the accessibility of biologics across APAC, generating high‐quality clinical evidence and standardizing scoring systems/assessment tools for optimal management of GPP. Regions where dermatologists are hesitant to prescribe biologics warrant investigation to understand the reasons and mitigate barriers to optimal treatment use.

**TABLE 4:** Approval and availability of biologics for GPP in the APAC region.

**Class**	Approved^a^	Available for off‐label use^b^
TNF‐α inhibitor	Adalimumab (Japan)	Adalimumab
	Infliximab (Japan)	Infliximab
	Certolizumab pegol (Japan)	Certolizumab pegol
		Etanercept
IL‐17/IL‐17R inhibitor	Secukinumab (Japan)	Secukinumab
	Ixekizumab (Japan)	Ixekizumab
	Brodalumab (Japan, Thailand, Taiwan)	Brodalumab
	Bimekizumab (Japan)	Bimekizumab
IL‐23 inhibitor	Guselkumab (Japan, Taiwan)	Guselkumab
	Risankizumab (Japan)	Risankizumab
		Tildrakizumab
IL‐36 inhibitor	Spesolimab (Australia, China, Japan, Korea, Philippines, Singapore, Taiwan)	Spesolimab
IL12/IL‐23 inhibitor		Ustekinumab

Abbreviations: APAC, Asia‐Pacific; GPP, generalized pustular psoriasis; IL, interleukin; TNF, tumor necrosis factor.

^a^ As of April 23, 2024.

^b^ Availability for off‐label use may vary in individual countries.

## 4 DISCUSSION

There has been a lack of guidelines on the classification, diagnosis, and management of GPP specific to the APAC region. This modified Delphi study, a collaborative effort among 20 esteemed experts from 10 APAC countries, represents a pivotal stride toward addressing this gap. The primary focus was on deriving consensus‐based recommendations tailored to the unique nuances of GPP management within the APAC context. Despite the consensus on a substantial 106 statements, it is crucial to acknowledge the diversities in clinical practices and treatment availabilities that led to some disagreements among the experts.

Notably, the assertion that GPP can manifest at any age,^3^ albeit less commonly in children,^11^ encountered a lower level of agreement. This highlights the complexity of understanding the epidemiology of GPP in the APAC region and emphasizes the need for further research to elucidate these dynamics. Additionally, statements regarding liver and renal involvement, which are commonly recognized as complications,^1^, ^3^, ^9^ also failed to achieve consensus. This discrepancy underscores the heterogeneity within the APAC patient population, necessitating a nuanced approach to GPP management in this region. However, a remarkable consensus was reached on the definition and severity classification of GPP flares, providing a practical framework for treatment decisions.

Regarding the diagnosis and severity assessment of GPP, the experts demonstrated pragmatism by not deeming biopsy or genetic testing mandatory for GPP diagnosis. This decision reflects acknowledgement of the limited accessibility to these diagnostic modalities within the region. Additionally, while tools like Generalized Pustular Psoriasis Physician Global Assessment (GPPASI) and Clinical Global Impression (CGI) were considered valuable for clinical trials, the experts exercised practical discretion by not recommending them for routine clinical practice, opting for more feasible alternatives like BSA, GPPGA, DLQI, and Pain‐VAS.

A remarkable consensus emerged on treatment goals, mirroring recent global and national trends, and emphasizing the urgency of rapidly resolving cutaneous and systemic signs and symptoms.^5^, ^7^, ^36^, ^37^ The stipulation that the treatment goal should include rapid fever clearance as soon as possible, preferably within 1 week, pustule clearance within 2 weeks, and skin clearance within 4 weeks highlights the imperative to address the multifaceted impact of GPP swiftly.

The discourse on treatment strategies, particularly the preference for biologics in managing severe flares, resonates with global perspectives, advocating timely access to rapidly acting and highly effective therapeutic agents, as delay may increase the risk of complications and mortality of this severe disease.^5^, ^36^, ^37^, ^38^, ^39^ Several biologics reported early response, such as spesolimab, infliximab, and secukinumab, which resulted in improvements as early as 24–72 h.^40^, ^41^, ^42^, ^43^, ^44^, ^45^ However, only spesolimab has robust evidence demonstrating its efficacy and safety in the continuous treatment of GPP (rapidly resolving and preventing further GPP flares), as demonstrated by the randomized, placebo‐controlled EFFISAYIL^®^ 1 and EFFISAYIL^®^ 2 trials.^41^, ^46^

Besides biologics, acitretin, cyclosporine, and methotrexate are recommended to treat GPP when biologics are not available, although the evidence for their efficacy is weak. The experts did not reach a consensus on using etretinate as an alternative to acitretin as a first‐line treatment for GPP flares, primarily because etretinate is currently available only in Japan. Nonetheless, etretinate is recognized as an acceptable alternative when acitretin is not accessible. The experts cautioned against the use of systemic corticosteroids, as they are known triggers of GPP flares,^1^ and their use as monotherapy for flares is associated with high mortality.^47^ They may be considered in pregnant patients where other treatment options are limited; however, abrupt withdrawal should be avoided to prevent triggering flares.

Findings from this modified Delphi panel study were summarized into treatment algorithms, which could guide physicians in the region on the optimal treatment approach when managing patients with GPP. Recognizing the potentially life‐threatening nature of GPP, the emphasis on optimal and timely care underlines the urgency in delivering rapidly effective interventions.

### 4.1 Strength and limitations

This study possesses both strengths and limitations. The engagement of a panel comprising internationally recognized GPP experts, with extensive experience in GPP management within the APAC region, underscores the robustness of the recommendations. The wealth of clinical knowledge and expertise contributed by these experts ensures that the developed consensus recommendation on the management of GPP is firmly rooted in both practical insights and contemporary evidence. However, a notable limitation is the relatively small number of APAC experts due to the rarity of GPP. Moreover, selection bias and voluntary participation in this modified Delphi study may introduce unintentional biases, as individuals who choose to engage may possess distinct perspectives or experiences compared to those who opt not to participate. Future research should aim to include a more diverse and larger pool of experts to mitigate selection bias and improve the generalizability and applicability of the guidelines.

## 5 CONCLUSION

This modified Delphi panel study contributes invaluable consensus‐based recommendations for GPP management in the APAC region and highlights the complexities and regional diversities that require ongoing research and adaptive clinical approaches. The optimal management of GPP flares necessitates an approach based on flare severity and individual patient considerations within the diverse APAC region.

## FUNDING INFORMATION

The study was supported and funded by Boehringer Ingelheim.

## CONFLICT OF INTEREST STATEMENT

S.E.C. declared paid activities as an advisor, speaker or consultant for AbbVie, Almirall, Boehringer Ingelheim, Eli Lilly, Janssen, Novartis, and Pfizer. P.A.F. declared receiving grant support from AbbVie, Amgen, Bristol Myers Squibb, Celgene, Eli Lilly, Galderma, Janssen, Leo Pharma, Merck, Novartis, Pfizer, Sanofi, Sun Pharma, and UCB; travel grants from AbbVie, Eli Lilly, Galderma, Janssen, Leo Pharma, Merck, Novartis, Pfizer, Roche, Sanofi, and Sun Pharma; and declared activities as an investigator for AbbVie, Amgen, Arcutis, Argenx, ASLAN, AstraZeneca, Boehringer Ingelheim, Botanix, Bristol Myers Squibb, Celgene, Celtaxsys, CSL, Cutanea, Dermira, Eli Lilly, Evelo Biosciences, Galderma, Genentech, Geneseq, GlaxoSmithKline, Hexima, Incyte, Janssen, Kymab, Leo Pharma, Merck, Novartis, Pfizer, Regeneron, Reistone, Roche, Sanofi, Sun Pharma, Takeda, Teva, UCB, Valeant, and ZaiLab; an advisory board member for AbbVie, Amgen, Aslan, Boehringer Ingelheim, Bristol Myers Squibb, Celgene, Eli Lilly, Galderma, GlaxoSmithKline, Janssen, Leo Pharma, Mayne Pharma, Novartis, Pfizer, Sanofi, Sun Pharma, UCB, and Valeant; a consultant for Aslan, Bristol Myers Squibb, Eli Lilly, Galderma, GenesisCare, Hexima, Janssen, Leo Pharma, Mayne Pharma, MedImmune, Novartis, Pfizer, Roche, and UCB; as a speaker or honoraria recipient from AbbVie, Amgen, Bristol Myers Squibb, Celgene, Eli Lilly, Galderma, GlaxoSmithKline, Janssen, Leo Pharma, Merck, Novartis, Pfizer, Roche, Sanofi, Sun Pharma, and Valeant. P.A. declared receiving honoraria or payment from Boehringer Ingelheim, Pfizer, Eli Lilly, Novartis and Janssen, and support for attending meetings and/or travel from Pfizer, Eli Lilly, and Novartis. H.F. has received honoraria or fees for serving on advisory boards, as a speaker and as a consultant, as well as grants as an investigator from AbbVie, Amgen, Boehringer Ingelheim, Celgene, Eisai, Eli Lilly, Janssen, Kyowa Kirin, LEO Pharma, Maruho, Mitsubishi‐Tanabe, Novartis, Sanofi, Sun Pharma, Taiho, Torii, UCB, and Ushio. H.F. is an Editorial Board member of *Journal of Dermatology* and to minimize bias, he was excluded from all editorial decision‐making related to the acceptance of this article for publication. S.J.J. declared activities as an advisor, speaker, or consultant for and/or received research grants from AbbVie, Boehringer Ingelheim, Bristol Myers Squibb, Celltrion Healthcare, Daewoong, Eli Lilly and Company, Green Cross Laboratories, Janssen Pharmaceuticals, Kolon Pharma, LEO Pharma, Novartis, Pfizer, Sanofi, UCB, and Yuhan. C.T. has served as a speaker or consultant for Janssen, AbbVie, Novartis, Amgen, and Boehringer Ingelheim. A.M.A. has received honoraria for serving as advisor and speaker for AbbVie, Boehringer Ingelheim, Janssen, Leo Pharma, Novartis, Pfizer, Sanofi and ZP Therapeutics. A.M.A. is also the principal investigator for clinical trials funded by Boehringer Ingelheim. C.H.B. declared paid activities as an advisor, speaker or consultant and/or received research grants from AbbVie, Boehringer Ingelheim, Bristol Myers Squibb, Celltrion, Eli Lilly, Janssen, Kolon Pharma, Leo Pharma, Novartis, Pfizer, and UCB. M.L.F. has received educational grants and honoraria or fees for serving as a member of advisory board, speaker for Johnson & Johnson, Novartis, Zuellig Pharma, and Leo Pharma. H.Y.H. has conducted clinical trials while serving as a principal investigator for AbbVie, Bristol‐Myers Squibb, Galderma, Eli Lilly, Novartis, Janssen‐Cilag Pharmaceutical, and Pfizer; received honoraria for serving as an advisory board member for Pfizer, AbbVie, and Celgene; and received speaking fees from AbbVie, Eli Lilly, and Novartis. A.M. has received research grants, consultancy fees, and/or speaker's fees from AbbVie, Amgen, Boehringer Ingelheim, Bristol‐Myers Squibb, Eisai, Eli Lilly Japan, Janssen Pharmaceutical, Kyowa Kirin, LEO Pharma, Maruho, Minophagen Pharmaceutical, Mitsubishi Tanabe Pharma, Nippon Kayaku, Novartis, Pfizer Japan, Sun Pharma Japan, Taiho Pharmaceutical, Torii Pharmaceutical, UCB Japan, and Ushio. H.H.O. has served as a speaker, advisory board member, and researcher for AbbVie, Galderma, Janssen and Novartis. She has also been a clinical investigator for Pfizer and Sanofi, advisory board member for Amgen as well as a speaker and advisory board member for Boehringer Ingelheim and Eli Lilly. P.F.P. has served on advisory boards for AbbVie, Amgen, Boehringer Ingelheim, Bristol Myers Squib, Celgene, Janssen, LEO Pharma, Eli Lilly and Company, Merck, Merck Sharp & Dohme, Novartis, Roche, Sanofi, and Sun Pharma; has given educational lectures for AbbVie, Amgen, Avene, Eli Lilly, Galderma, Janssen, La Roche‐Posay, LEO Pharma, Merck, Novartis, Pfizer, Roche, Sanofi, Schering Plough, Sun Pharma, and UCB Pharma; has conducted clinical trials for AbbVie, Amgen, Arena, AstraZeneca, Boehringer Ingelheim, Bristol Myers Squibb, CSL, Dermira, Eisai, Eli Lilly and Company, Galderma, GlaxoSmithKline, Janssen, Jiangsu Hengrui, Kyowa Hakko Kirin, LEO Pharma, miRagen, Novartis, OncoSec, Pfizer, Regeneron, Roche, Sun Pharma, UCB Pharma, and Xoma. L.S. declared paid activities as an advisor/speaker for AbbVie, Boehringer Ingelheim, DKSH, Eli Lilly, Janssen, Leo Pharma, MSD, Novartis, Sanofi, and Zuellig Pharma. T.F.T. has conducted clinical trials or received honoraria for serving as a consultant for AbbVie, AnaptysBio, Boehringer Ingelheim, Bristol‐Myers Squibb, Celgene, Eli Lilly, Galderma, GSK, Janssen‐Cilag, Leo Pharma, Merck Sharp & Dohme, Novartis International, Pfizer, PharmaEssentia, Sanofi, Sun Pharma, and UCB Pharma. Y.S., D.L.H., T.G.K., N.R., and S.R. did not have conflicts of interests to disclose.

## 6 CONFLICT OF INTEREST STATEMENT

Hideki Fujita is an Editorial Board member of *Journal of Dermatology* and a co‐author of this article. To minimize bias, he was excluded from all editorial decision‐making related to the acceptance of this article for publication.

## Supporting information


Data S1.

## Data Availability

To ensure independent interpretation of clinical study results and enable authors to fulfill their role and obligations under the ICMJE criteria, Boehringer Ingelheim grants all external authors access to relevant clinical study data. In adherence with the Boehringer Ingelheim Policy on Transparency and Publication of Clinical Study Data, scientific and medical researchers can request access to clinical study data, typically 1 year after the approval has been granted by major Regulatory Authorities or after termination of the development program. Researchers should use the https://vivli.org/ link to request access to study data and visit https://www.mystudywindow.com/msw/datasharing for further information.
