# Supplementary material for: Asia‐Pacific consensus recommendations on the management of generalized pustular psoriasis
Source: J Dermatol. 2024 Oct 10;51(12):1579–95. doi: 10.1111/1346-8138.17471 (PMC11624156; doi:10.1111/1346-8138.17471)
Supplement: Supplementary file 1 — Data S1. [file JDE-51-1579-s001.docx]

# Supplementary INFORMATION

# Supplementary methods

Following de-duplication, publications identified through the searches were screened for inclusion or exclusion by two independent reviewers. Studies eligible for inclusion were those conducted in patients with GPP and included randomized controlled trials (RCTs); observational studies; meta-analyses; non-RCTs; reviews; systemic reviews; guidelines; consensus papers; and case reports/series. All included publications were in the English language; there were no geographical restrictions. In vitro/animal studies, protocol papers, or comment articles were excluded from the SLR.

For treatment-related statements, an “I don’t have relevant experience” option was included in the answers, and the percentage of agreement was calculated after excluding the number of experts who selected this option.

# Figure S1. PRISMA diagram of systematic literature review using MEDLINE database.


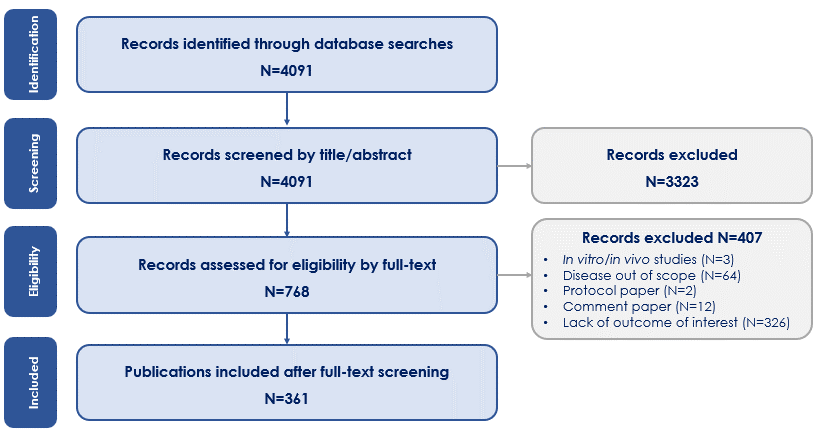


# Table S1. Systematic literature search strategy and number of hits.

| **Domain 1. Definition and clinical course of GPP** | | | | |
| --- | --- | --- | --- | --- |
| String No. | Search query | | No. of literature hits | |
| 1 | ((Generalized pustular psoriasis) OR (GPP) OR (pustular psoriasis) OR (von Zumbusch)) AND ((classification) OR (definition)) | | 221 | |
| 2 | ((Generalized pustular psoriasis) OR (GPP) OR (pustular psoriasis) OR (von Zumbusch)) AND ((signs) OR (symptom) OR (skin symptom) OR (systemic symptom)) | | 1451 | |
| 3 | ((Generalized pustular psoriasis) OR (GPP) OR (pustular psoriasis) OR (von Zumbusch)) AND ((clinical course) OR (disease course)) | | 172 | |
| 4 | ((Generalized pustular psoriasis) OR (GPP) OR (pustular psoriasis) OR (von Zumbusch)) AND (flare) | | 103 | |
| 5 | ((Generalized pustular psoriasis) OR (GPP) OR (pustular psoriasis) OR (von Zumbusch)) AND ((trigger) OR (precipitating factor) OR (risk factor) OR (predispos* factor)) | | 284 | |
| 6 | ((Generalized pustular psoriasis) OR (GPP) OR (pustular psoriasis) OR (von Zumbusch)) AND ((prognosis) OR (comorbidity) OR (concomitant psoriatic arthritis) OR (concomitant arthritis) OR (complication)) | | 856 | |
|  | **#1 OR #2 OR #3 OR #4 OR #5 OR #6** | | 1992 | |
| **Domain 2. Diagnostic criteria for GPP** | | | |  |
| String No. | Search query | No. of literature hits | |  |
| 1 | ((Generalized pustular psoriasis) OR (GPP) OR (pustular psoriasis) OR (von Zumbusch)) AND ((diagnos*) OR ((guideline) OR (consensus))) | 927 | |  |
| 2 | ((Generalized pustular psoriasis) OR (GPP) OR (pustular psoriasis) OR (von Zumbusch)) AND (diagnos*) AND ((clinical) OR (histopatholog*) OR (histology*) OR (laboratory test) OR (laboratory evaluation) OR (biomarker)) | 461 | |  |
| 3 | ((Generalized pustular psoriasis) OR (GPP) OR (pustular psoriasis) OR (von Zumbusch)) AND ((genetic screening) OR (genetic testing) OR (mutation) OR (polymorphism)) | 317 | |  |
| 4 | ((Generalized pustular psoriasis) OR (GPP) OR (pustular psoriasis) OR (von Zumbusch)) AND (differential diagnosis) | 205 | |  |
|  | **#1 OR #2 OR #3 OR #4** | 1171 | |  |

| **Domain 3. Treatment outcomes, goals and monitoring measures for GPP** | | |
| --- | --- | --- |
| String No. | Search query | No. of literature hits |
| 1 | ((Generalized pustular psoriasis) OR (GPP) OR (pustular psoriasis) OR (von Zumbusch)) AND ((outcome) OR (target) OR (objective) OR (response) OR (success) OR (assessment) OR (assess*) OR (measurement) OR (measur* endpoint) OR (evaluation) OR (evaluat*)) | 2396 |
| 2 | ((Generalized pustular psoriasis) OR (GPP) OR (pustular psoriasis) OR (von Zumbusch)) AND ((assessment) OR (assess*) OR (evaluation) OR (evaluat*) OR (monitor*)) AND ((severity) OR (progression) OR (complication) OR (comorbidity)) | 348 |
|  | **#1 OR #2** | 2317 |

| **Domain 4. Optimal management strategies and clinical practices** | | |
| --- | --- | --- |
| String No. | Search query | No. of literature hits |
| 1 | ((Generalized pustular psoriasis) OR (GPP) OR (pustular psoriasis) OR (von Zumbusch)) AND ((treatment) OR (management)) AND ((guideline) OR (consensus)) | 60 |
| 2 | ((Generalized pustular psoriasis) OR (GPP) OR (pustular psoriasis) OR (von Zumbusch)) AND ((treatment) OR (therap*) OR (systemic) OR (medication) OR (management) OR (practice) OR (acrodermatitis continua) OR (acrodermatitis)) | 2683 |
| 3 | ((Generalized pustular psoriasis) OR (GPP) OR (pustular psoriasis) OR (von Zumbusch)) AND ((biologics) OR (biological agents)) | 1717 |
|  | **#1 OR #2 OR #3** | 3422 |

Abbreviation: GPP, generalized pustular psoriasis.

# Table S2. Demographics of the Delphi panellists.

| **Characteristic** | **Panellists (N=20)** |
| --- | --- |
| Age, mean (range) | 53.4 (40–67) |
| Sex, n (%) |  |
| Female | 9 (45) |
| Male | 11 (55) |
| Years of experience, mean (range) | 24.4 (8–39) |
| Hospital setting, n (%) |  |
| Academic | 5 (25) |
| Public | 7 (35) |
| Private | 2 (10) |
| Academic & Public | 4 (20) |
| Academic & Private | 1 (5) |
| Public & Private | 1 (5) |
| Clinical setting, n (%) |  |
| Inpatient | 1 (5) |
| Outpatient | 3 (15) |
| Inpatient & Outpatient | 16 (80) |
| No. of GPP patients seen in the past 5 years, mean (range) | 39.0 (5–250) |

Abbreviation: GPP, generalized pustular psoriasis.

# Table S3. Statements that were revised following the Delphi survey.

| **1. DEFINITION AND CLINICAL COURSE** | **Consensus** | | |
| --- | --- | --- | --- |
| **Epidemiology of GPP** | **Round 1 (%)** | | **Round 2 (%)** |
| *Statement in Delphi Survey:*  GPP onset is less common in children than in adults. | 67% | | NA |
| *Revised statement:*  GPP is less common in children. | NA | | 74% |
| **Classification of GPP** | **Round 1 (%)** | **Round 2 (%)** | |
| *Statement in Delphi Survey:*  Patients with GPP can present with or without concomitant plaque psoriasis. | 92% | NA | |
| *Revised statement:*  Patients with GPP may or may not have associated plaque psoriasis. | NA | 100% | |
| **Flare definition and clinical course** | **Round 1 (%)** | | **Round 2 (%)** |
| *Statement in Delphi Survey:*  Most patients have clear skin between flares. | 42% | | NA |
| *Revised statement:*  Patients with GPP may have clear skin between flares, except in the setting of concomitant plaque psoriasis. | NA | | 100% |
| *Statement in Delphi Survey:*  Most patients have residual disease between flares. | 75% | | NA |
| *Revised statement:*  Patients with GPP may have residual disease such as erythema with pustules between flares. | NA | | 84% |
| **3. TREATMENT OUTCOMES, GOALS, AND MONITORING MEASURES FOR GPP** | | | |
| **Short-term/flare-phase treatment goals** | **Round 1 (%)** | | **Round 2 (%)** |
| *Statement in Delphi Survey:*  Treatment goal should be pustular clearance and resolution of fever within 1–2 weeks, with skin clearance within four weeks. | 75% | | NA |
| *Revised statement:*  Treatment goal should be clearance of pustules and resolution of fever as soon as possible, preferably within one week, with skin clearance within four weeks. | NA | | 100% |
| **Long-term treatment goals** | **Round 1 (%)** | | **Round 2 (%)** |
| *Statement in Delphi Survey*:  Due to the substantial emotional burden of GPP beyond the physical discomfort of skin lesions; improving patients’ quality of life is the ultimate goal of the treatment. | 75% | | NA |
| *Revised statement*:  Due to the substantial emotional burden of GPP beyond the physical discomfort of skin lesions; improving patients’ quality of life through effective treatments is an important treatment goal. | NA | | 100% |
| **4. OPTIMAL MANAGEMENT STRATEGIES AND CLINICAL PRACTICES** | | | |
| **Treatment strategies** | **Round 1 (%)** | | **Round 2 (%)** |
| *Statement in Delphi Survey*:  Biologics are the preferred treatment of choice when managing acute flares, if accessible. | 75% | | NA |
| *Revised statement*:  Currently, biologics are the preferred treatment of choice when managing acute flares, if accessible. | NA | | 100% |
| **Systemic treatment for flare and maintenance phase** | **Round 1 (%)** | | **Round 2 (%)** |
| **Flare phase: Preferred therapy** |  | |  |
| *Statement in Delphi Survey*:  High-dose acitretin is recommended as first-line treatment to manage acute flares. | 55%* | | NA |
| *Revised statement*:  High-dose acitretin is recommended as first-line treatment to manage acute flares when biologics are not available/accessible. | NA | | 84% |
| *Statement in Delphi Survey*:  High-dose cyclosporine is recommended as first-line treatment to manage acute flares. | 75%* | | NA |
| *Revised statement*:  High-dose cyclosporine is recommended as first-line treatment to manage severe acute flares when biologics are not available/accessible. | NA | | 95% |
| *Statement in Delphi Survey*:  IL-17 inhibitors are recommended as first-line treatment to manage acute flares. | 60%* | | NA |
| *Revised statement*:  IL-17 inhibitors can be considered for managing acute flares if other preferred therapies are not accessible. | NA | | 89% |
| *Statement in Delphi Survey*:  High-dose acitretin is recommended as second-line treatment to manage acute flares. | 58%* | | NA |
| *Revised statement*:  High-dose acitretin can be considered as second-line treatment to manage acute flares. | NA | | 88%* |
| **Maintenance phase: Preferred therapy** |  | |  |
| *Statement in Delphi Survey*:  Low-dose cyclosporine is the recommended treatment for maintenance phase. | 67%* | | NA |
| *Revised statement*:  Low-dose cyclosporine can be used for maintenance phase. | NA | | 70% |
| *Statement in Delphi Survey*:  Methotrexate is the recommended treatment for maintenance phase. | 75%* | NA | |
| *Revised statement*:  Methotrexate can be used for maintenance phase. | NA | 95% | |
| *Statement in Delphi Survey*:  IL-36 inhibitors are the recommended treatment for maintenance phase. | 60%* | NA | |
| *Revised statement*:  IL-36 inhibitors can be used for maintenance phase. | NA | 83% | |
| *Statement in Delphi Survey*:  IL-17 inhibitors are the recommended treatment for maintenance phase. | 79%* | NA | |
| *Revised statement*:  IL-17 inhibitors can be used for maintenance phase. | NA | 100% | |
| *Statement in Delphi Survey*:  IL-23 inhibitors are the recommended treatment for maintenance phase. | 50%* | | NA |
| *Revised statement*:  IL-23 inhibitors can be used for maintenance phase. | NA | | 94%* |
| *Statement in Delphi Survey*:  TNF-α inhibitors are the recommended treatment for maintenance phase. | 56%* | | NA |
| *Revised statement*:  TNF-α inhibitors can be used for maintenance phase. | NA | | 69%* |
| **Biologic treatments for the management of GPP** | **Round 1 (%)** | **Round 2 (%)** | |
| *Statement in Delphi Survey*:  TNF-α inhibitors are not recommended for patients with a history of tuberculosis. | 83%* | NA | |
| *Revised statement*:  TNF-α inhibitors are not recommended for patients with active or latent tuberculosis (TB); they can be used in treated TB or one month after commencement of treatment for latent TB. | NA | 100%* | |
| **Non-biologic treatments for the management of GPP** | **Round 1 (%)** | | **Round 2 (%)** |
| *Statement in Delphi Survey*:  If the patient’s condition improves within 2–3 weeks of starting systemic treatments in the acute phase (pustule improvement, no appearance of new lesions), the dose of non-biologic treatment should be tapered according to clinical response. | 75%* | | NA |
| *Revised statement*:  If the patient’s condition improves within 2–4 weeks of starting systemic treatments in the acute phase (pustule improvement, no appearance of new lesions), the dose of non-biologic treatment can be tapered gradually according to clinical response. Abrupt and/or early tapering may result in flares and suboptimal disease control. | NA | | 95% |
| **Management of childhood GPP** | **Round 1 (%)** | | **Round 2 (%)** |
| *Statement in Delphi Survey*:  Acitretin is recommended for the management of GPP in pediatric patients. | 70%* | | NA |
| *Revised statement*:  Acitretin can be used for the management of GPP in pediatric patients when biologics are not available/accessible. | NA | | 100% |
| **Management of GPP in pregnancy** | **Round 1 (%)** | | **Round 2 (%)** |
| *Statement in Delphi Survey*:  Low-dose systemic corticosteroids are recommended for the management of GPP in pregnant patients. | 67%* | | NA |
| *Revised statement*:  Low-dose systemic corticosteroids may be considered for the management of GPP in pregnant patients if other treatment options fail/are not available. | NA | | 83% |
| *Statement in Delphi Survey*:  TNF-α inhibitors are recommended for the management of GPP in pregnant patients. | 60%* | NA | |
| *Revised statement*:  TNF-α inhibitors^†^ should be considered carefully when treating GPP in pregnant patients based on the risk-benefit profile for individual patients. | NA | 100%* | |
| *Statement in Delphi Survey*:  Dermatologists should work closely with OB-GYNs to frequently monitor fetal heartbeat variability and low body weight as indications of placental insufficiency to prevent any negative outcomes. | 92%* | NA | |
| *Revised statement*:  Dermatologists should work closely with OB-GYNs to prevent any negative outcome. | NA | 100% | |
| **Holistic management for patients with GPP** | **Round 1 (%)** | **Round 2 (%)** | |
| *Statement in Delphi Survey*:  Patients should avoid stress and trauma, especially as stress is considered a trigger for GPP flares. | 100%* | NA | |
| *Revised statement*:  Patients should avoid smoking and trauma, and manage stress. | NA | 85% | |

*Number of experts who selected ‘I don’t have relevant experience’ is excluded when calculating the consensus.
^†^Certolizumab pegol is the preferred TNF-α inhibitor.
Abbreviations: GPP, generalized pustular psoriasis; IL, interleukin; OB-GYN, obstetrician-gynecologist; TB, tuberculosis; TNF, tumor necrosis factor.

# Table S4. New statements that were developed following the Delphi survey.

| **1. DEFINITION AND CLINICAL COURSE** | **Consensus** | | |
| --- | --- | --- | --- |
| **Flare definition and clinical course** | **Round 1 (%)** | | **Round 2 (%)** |
| GPP flares that affect at least 10% of the body surface area can be defined as severe GPP.* | NA | | 100% |
| GPP flares that affect less than 3% of the body surface area with concomitant systemic symptoms, can be defined as severe GPP.* | NA | | 90% |
| GPP flares that affect <3% of body surface area without concomitant systemic symptoms can be defined as mild GPP.^†^ | NA | | 100% |
| GPPGA total score <3 can be defined as mild GPP.^†^ | NA | | 94% |
| GPPGA pustulation score <3 can be defined as mild GPP.^†^ | NA | | 100% |
| **Complications, comorbidities, and prognosis** | **Round 1 (%)** | **Round 2 (%)** | |
| Obesity is a common comorbidity among Asian patients with GPP.* | NA | 28% | |
| **2. DIAGNOSIS OF GPP** | | | |
| **Medical and family history** | **Round 1 (%)** | | **Round 2 (%)** |
| GPP is distinct from GPP with plaque psoriasis.* | NA | | 74% |
| **3. TREATMENT OUTCOMES, GOALS, AND MONITORING MEASURES FOR GPP** | | | |
| **Short-term/flare-phase treatment goals** | **Round 1 (%)** | | **Round 2 (%)** |
| Treatment goal should be clearance of pustules and resolution of fever as soon as possible, preferably within two weeks, with skin clearance within four weeks.* | NA | | 32% |
| **4. OPTIMAL MANAGEMENT STRATEGIES AND CLINICAL PRACTICES** | | | |
| **Systemic treatment for flare and maintenance phase** | **Round 1 (%)** | | **Round 2 (%)** |
| **Flare phase: Preferred therapy** |  | |  |
| What is the preferred or recommended maintenance treatment for mild GPP?^†^ |  | |  |
| - Acitretin | NA | | 89% |
| - Cyclosporine | NA | | 67% |
| - Methotrexate | NA | | 61% |
| - Topical steroids | NA | | 61% |
| - Other treatments | NA | | 17% |
| **Maintenance phase: Preferred therapy** |  | |  |
| What is the preferred or recommended maintenance treatment for mild GPP?^†^ | NA | |  |
| - Acitretin | NA | | 85% |
| - Cyclosporine | NA | | 30% |
| - Methotrexate | NA | | 70% |
| - Topical steroids | NA | | 55% |
| - Other treatments | NA | | 20% |
| **Management of childhood GPP** | **Round 1 (%)** | | **Round 2 (%)** |
| Acitretin is recommended for the management of acute GPP flares in children.^†^ | NA | | 94% |
| Spesolimab is recommended for the management of acute GPP flares in children.^†^ | NA | | 27% |
| Spesolimab may be considered for the management of acute GPP flares in children.^†^ | NA | | 79% |
| IL-36 inhibitors may be considered for the management of acute GPP flares in children who failed the standard treatments.^†^ | NA | | 100% |

*New statements that were developed based on the Delphi survey (Round 1) feedback.
^†^New statements that were developed during the virtual consensus meeting (Round 2).
Abbreviations: GPP, generalized pustular psoriasis; GPPGA, Generalized Pustular Psoriasis Physician Global Assessment; IL, interleukin.
